# Transcutaneous vagus nerve stimulation in sleep disorders: current evidence and future directions

**DOI:** 10.3389/fpain.2026.1772166

**Published:** 2026-07-16

**Authors:** Frédéric Roche, Mathilde Monier, Lytissia Mouhli Gasmi, Vincent Pichot

**Affiliations:** 1 Physiologie Clinique et de L'Exercice, CHU Saint Etienne, Saint Etienne, France; 2 CRNL Fluid Team, Université Jean Monnet Saint Etienne, Saint Etienne, France; 3 Sainbiose Inserm U1059, Ecole des Mines, Université Jean Monnet Saint Etienne, Saint Etienne, France

**Keywords:** autonomic nervous system, parasympathetic activity, sleep disorders, tVNS, vagal stimulation

## 1 Introduction

Transcutaneous vagus nerve stimulation (tVNS) applied to the auricular region has emerged as a promising non-invasive neuromodulatory technique for various neuropsychiatric conditions (1). Sleep disorders represent a major public health burden, and autonomic nervous system dysregulation is recognized as a key pathophysiological driver across multiple sleep pathologies (2). Given the known effects of vagal tone on sleep-wake regulation (3), tVNS holds theoretical and emerging clinical potential in this domain.

Terminology is important: throughout this article, we use “tVNS” as the broad term for all forms of transcutaneous (non-invasive) vagal stimulation, and “taVNS” (transcutaneous auricular VNS) when specifically referring to auricular approaches. The tragus and cymba concha are distinct anatomical sites with different afferent nerve densities and potentially different therapeutic profiles, and are specified for each study. Invasive cervical VNS (implanted device) is an entirely different intervention with distinct safety implications and is explicitly distinguished throughout.

The objective of this opinion article is to provide a structured synthesis of available evidence regarding tVNS across different sleep pathologies, to critically discuss methodological limitations of the existing literature, and to identify priority areas for future research. As this is an opinion article based on a selective narrative literature review rather than a systematic review, the conclusions represent expert perspectives and informed hypotheses rather than definitive evidence-based recommendations.

## 2 Methods

We conducted a selective narrative literature search of PubMed/MEDLINE and Cochrane Library databases from inception to March 2026. Search terms included: “transcutaneous vagus nerve stimulation”, “transcutaneous auricular vagus nerve stimulation”, “taVNS”, “tVNS”, combined with “sleep”, “insomnia”, “obstructive sleep apnea”, “restless legs syndrome”, “PTSD”, “sleep disorders”, “parasympathetic”, and “autonomic”. We prioritized randomized controlled trials (RCTs), pilot studies with polysomnographic endpoints, prospective cohort studies, and meta-analyses. Given the limited literature, case reports and animal studies were included when no human data were available. Studies were selected based on the authors' expert judgment of relevance and methodological quality; no formal risk-of-bias assessment was performed, consistent with the opinion article format. A total of 27 references were retained for this synthesis. All identified study limitations are discussed explicitly in the Results and Discussion sections.

## 3 Results

### 3.1 Anatomical and physiological rationale

**Figure 1:**
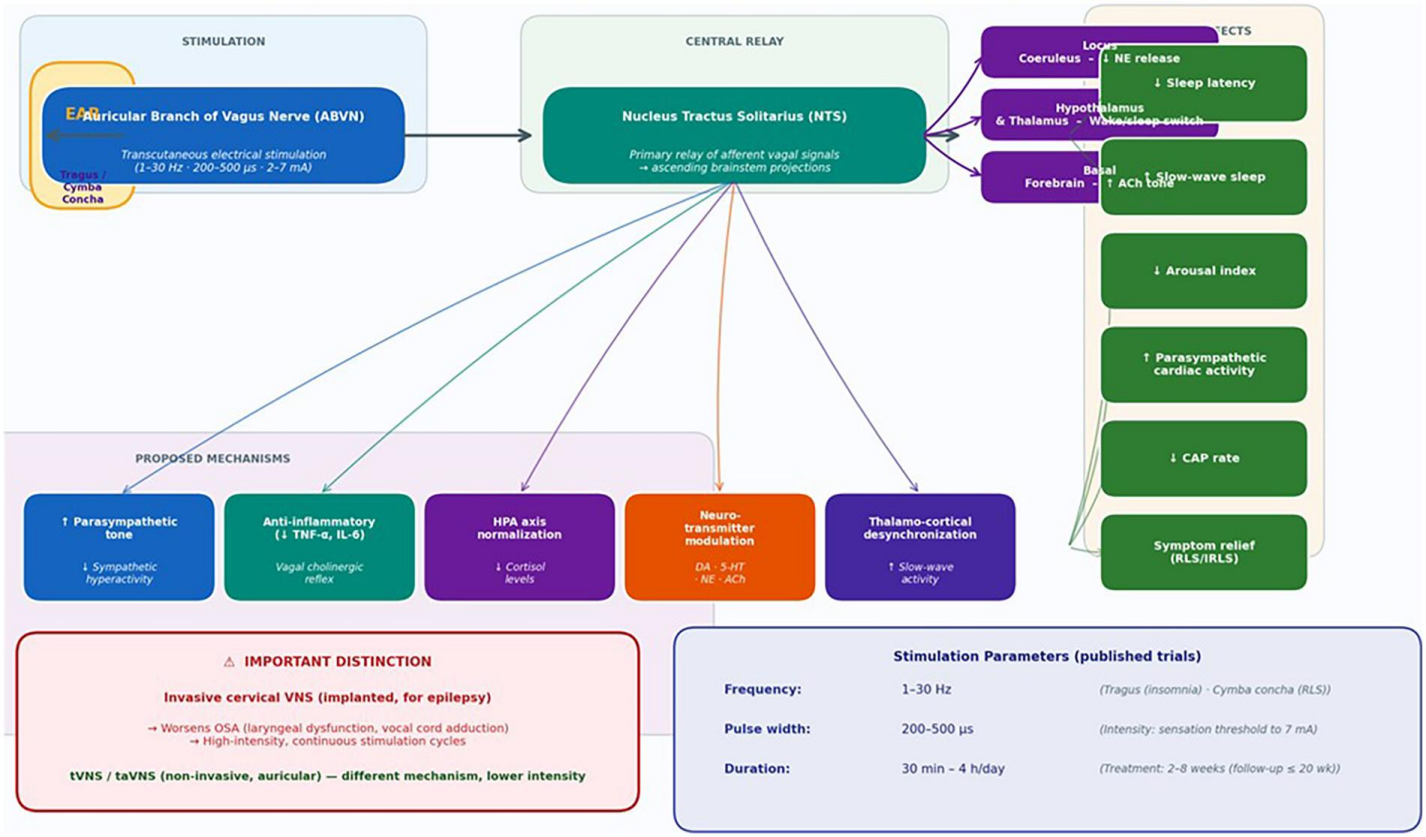
Proposed mechanisms of transcutaneous vagus nerve stimulation (tVNS) in sleep disorders. ABVN, auricular branch of vagus nerve; NTS, nucleus tractus solitarius; NE, norepinephrine; ACh, acetylcholine; DA, dopamine; 5-HT, serotonin; HPA, hypothalamic-pituitary-adrenal; CAP, cyclic alternating pattern; RLS, restless legs syndrome; IRLS, International RLS Rating Scale. Important: invasive cervical VNS (implanted) consistently worsens OSA through laryngeal dysfunction — mechanistically distinct from non-invasive auricular tVNS.

The auricular branch of the vagus nerve (ABVN) innervates the tragus, cymba concha, and external auditory canal, providing accessible cutaneous targets for non-invasive electrical stimulation (4). tVNS activates afferent vagal pathways projecting to the nucleus tractus solitarius (NTS), with subsequent connections to key sleep-regulatory brain regions including the locus coeruleus, hypothalamus, and thalamus (5). Physiologically, vagal activation enhances parasympathetic tone while modulating sympathetic activity, potentially addressing the autonomic imbalance characteristic of multiple sleep disorders (Figure 1) (6).

### 3.2 Clinical evidence by sleep disorder

A summary of all identified clinical studies is provided in Table 1 (separate file). The following subsections discuss the evidence by pathology.

**Table 1:** Summary of clinical evidence for tVNS in sleep disorders.

Sleep disorder	Study	Design/N	tVNS protocol	Primary outcome/Key results	Main limitations	Level of evidence^a^
Chronic insomnia	Zhang et al. 2024 (7)	RCT, DB, SC *n* = 72	Bilateral tragus 30 min BID × 8 wk	PSQI −4.2 pts (d = 1.2) ISI, anxiety, fatigue ↓ Follow-up 12 wk maintained	Single-center Modest sample	**Moderate**
High-altitude insomnia	Zhang et al. 2023 (8)	RCT *n* = 100	Tragus (parameters NR)	↓ Sleep latency ↑ SWS (PSG)	High-altitude ≠ chronic insomnia (hypoxia-driven arousal)	Low–moderate
Community adults (no clinical insomnia)	Jackowska et al. 2022 (9)	RCT *n* = 68	Tragus 4 h/day × 14 d	↓ PSQI (early & late phases)	Non-clinical population Short follow-up	Moderate
PTSD sleep disturbance	Bottari et al. 2024 (10)	Pilot, PSG *n* = 13	Tragus, 1 h at lights-out	↑ N3 (d = 0.23) ↓ REM (d = −0.48) ↓ CAP rate (d = −0.65)	***n*** **=** **13, no sham control Pilot only Single night**	**Very low**
OSA (animal model)	Guo et al. 2021 (11)	Animal (canine) No human trial	Low-level VNS Canine model	↓ AF inducibility ↓ Sympathetic tone	**Animal model only No human data available**	**Preclinical only**
OSA (invasive VNS – HARM)	Meta-analysis (12, 13)	Meta-analysis (invasive cervical VNS)	Implanted VNS (epilepsy device)	**27.3% new/worsened OSA (95% CI 15.1–41.5%)**	Invasive ≠ auricular tVNS Distinct mechanism/intensity	**High (for harm)**
Pharmacoresistant RLS	Hartley et al. 2023 (14) + 2024 (15)	Prospective, open-label *n* = 15 (8 wk + 6 mo)	Left cymba concha 2 Hz · 200 ms · 2–7 mA 1 h/wk → home	87% responders IRLS: 31.9 → 17.6 (d = 1.79) Maintained at 6 months QoL, depression, anxiety ↓	**No sham control Open-label *n*** **=** **15 only No active comparator**	**Low (no RCT)**
RLS – NPNS comparator	Charlesworth et al. 2023 (16)	RCT, SC *n* = 27	Bilateral peroneal nerve high-frequency stimulation	↓ RLS symptoms (spinal/gate mechanism)	Different mechanism from taVNS Not auricular vagal approach	Moderate (different intervention)

DB, double-blind; SC, sham-controlled; RCT, randomized controlled trial; PSG, polysomnography; PSQI, Pittsburgh Sleep Quality Index; ISI, Insomnia Severity Index; SWS, slow-wave sleep; CAP, cyclic alternating pattern; IRLS, International RLS Rating Scale; AF, atrial fibrillation; NR, not reported; QoL, quality of life; NPNS, noninvasive peroneal nerve stimulation; OSA, obstructive sleep apnea; RLS, restless legs syndrome; PTSD, post-traumatic stress disorder.

a Levels of evidence: High = multiple RCTs without major limitations; Moderate = RCT with limitations; Low = prospective uncontrolled; Very low = pilot/case series; Preclinical = animal data only.

#### 3.2.1 Chronic insomnia disorder

The currently most substantial body of evidence for tVNS in sleep medicine concerns insomnia. Three randomized controlled trials have been published. Zhang et al. (7) conducted a randomized, double-blind, sham-controlled trial (*n* = 72) demonstrating that 8 weeks of bilateral tragal tVNS (30 min twice daily) produced clinically meaningful reductions in Pittsburgh Sleep Quality Index (PSQI) scores (mean difference −4.2 points, 95% CI −5.9 to −2.6, Cohen's d = 1.2) compared with sham stimulation, with benefits persisting at 12-week follow-up and accompanied by reductions in Insomnia Severity Index scores, anxiety, and fatigue. A separate trial (*n* = 100) in high-altitude insomnia patients demonstrated objective improvements including shortened sleep latency and increased slow-wave sleep duration (SWS) (8). However, high-altitude insomnia involves hypoxia-driven arousal mechanisms distinct from chronic insomnia at sea level, limiting direct extrapolation. A third trial (*n* = 68) employing 4-hour daily tragal tVNS over 14 days in community-dwelling adults (without clinical insomnia) reported significant PSQI reductions (9). These three trials differ substantially in population, stimulation protocol, and sham design, precluding pooled effect estimation.

#### 3.2.2 PTSD-related sleep disturbance

A pilot polysomnographic study (*n* = 13) in veterans with PTSD examined 1-hour tragal tVNS at lights-out (10). Results showed modest increases in N3 sleep (Cohen's d = 0.23), reductions in REM sleep (d = −0.48), moderate decreases in cyclic alternating pattern (CAP) rate (d = −0.65), and increased parasympathetically-mediated cardiac autonomic activity during NREM sleep. These findings are preliminary and must be interpreted with extreme caution given the very small sample size (*n* = 13), uncontrolled design, and single-night measurement. They are hypothesis-generating and cannot support clinical recommendations.

#### 3.2.3 Obstructive sleep apnea

Evidence in OSA requires careful differentiation between invasive and non-invasive approaches. Invasive cervical VNS, used for refractory epilepsy, consistently worsens OSA: meta-analyses report a 27.3% (95% CI 15.1%–41.5%) prevalence of new or worsened sleep-disordered breathing post-implantation (12, 13), occurring through laryngeal dysfunction, vocal cord adduction, and central respiratory rhythm disruption (17). No controlled human trials have examined tVNS (auricular) effects on apnea-hypopnea index or oxygen desaturation. The only available data derive from a canine model of simulated OSA, in which low-level VNS reduced atrial fibrillation inducibility by ameliorating sympathetic hyperactivity (11). These animal data are hypothesis-generating only. Current evidence does not support tVNS as a treatment for OSA.

#### 3.2.4 Restless legs syndrome

Hartley et al. published two prospective studies of taVNS (left cymba concha, 2 Hz, 200-ms pulse width, 2–7 mA) in pharmacoresistant RLS. The first (*n* = 15, 8 weeks, 1 h/week) reported that 13/15 patients (87%) demonstrated clinically meaningful responses, with mean International RLS Rating Scale (IRLS) reduction from 31.9 ± 2.9 to 17.6 ± 10.4 (effect size d = 1.79) (14). The 6-month follow-up study reported maintenance of response in 13/15 patients with self-administered home therapy (IRLS 18.3 ± 10.0), alongside significant improvements in quality of life, depression, and anxiety (15). No serious adverse events occurred. Critical limitations are the open-label, uncontrolled design and very small sample size; the absence of a sham-control group is a major methodological gap that prevents firm conclusions about efficacy. A separate randomized sham-controlled trial of bilateral high-frequency noninvasive peroneal nerve stimulation (NPNS) also demonstrated RLS symptom reduction (16), supporting the concept of non-invasive neuromodulation for RLS through distinct spinal reflex mechanisms.

#### 3.2.5 Other sleep disorders

No controlled trials examine tVNS in circadian rhythm disorders, narcolepsy, parasomnias, or REM sleep behavior disorder. Theoretical rationales exist based on vagal modulation of relevant neurotransmitter systems (18), but clinical evidence is entirely lacking.

## 4 Discussion

### 4.1 Mechanisms of action

As illustrated in Figure 1, proposed mechanisms through which tVNS improves sleep include: (i) restoration of sympathovagal balance through enhanced parasympathetic tone and reduced sympathetic hyperactivity (19); (ii) modulation of wake-promoting neurotransmitters including norepinephrine and acetylcholine via locus coeruleus and basal forebrain projections (20); (iii) anti-inflammatory effects reducing pro-inflammatory cytokines (TNF-α, IL-6) implicated in sleep disruption (21); (iv) hypothalamic-pituitary-adrenal (HPA) axis normalization with reduced cortisol levels (22); and (v) direct effects on thalamo-cortical arousal systems promoting slow-wave activity. These mechanisms remain largely theoretical; few studies employ mechanistic endpoints (functional neuroimaging, autonomic biomarkers, inflammatory assays) to validate proposed pathways, and the relationships between specific stimulation parameters and therapeutic effects require systematic investigation (23).

### 4.2 Safety and tolerability

Published trials consistently report excellent safety profiles for auricular tVNS. Adverse effects are mild and transient, including local skin irritation, tingling sensations, and rare headache (7–9). No serious adverse events have been documented. tVNS appears to lack the respiratory complications associated with invasive cervical VNS, likely reflecting differences in stimulation intensity, anatomical targets, and intermittent vs. continuous stimulation. Nevertheless, systematic safety monitoring including polysomnographic respiratory parameters remains prudent in populations with comorbid respiratory disorders, as no adequately powered safety study in OSA patients exists for the auricular route.

### 4.3 Critical appraisal and methodological limitations

A critical reading of the available literature reveals several important methodological concerns that must be acknowledged before drawing clinical conclusions:

1. Protocol heterogeneity: Stimulation site (tragus vs. cymba concha), laterality (unilateral vs. bilateral), frequency (1–30 Hz), pulse width (200–500 µs), intensity, daily duration (30 min to 4 h), and treatment duration (2–8 weeks) vary substantially across trials, preventing any meaningful cross-study comparison or meta-analytic synthesis.
2. Sample size and power: Most trials are underpowered (*n* = 13 to *n* = 100), increasing risk of Type I errors, especially for secondary and exploratory outcomes.
3. Absence of active comparators: No trial compares tVNS with an established first-line treatment (cognitive behavioral therapy for insomnia [CBT-I], CPAP for OSA, dopaminergic therapy for RLS) (24). This limits clinical positioning.
4. Sham control validity: The adequacy of sham conditions (earlobe stimulation) for blinding remains uncertain; tactile perception from any auricular stimulation may be incompatible with true patient blinding.
5. Follow-up duration: Published follow-up periods rarely exceed 12–20 weeks. Long-term durability, maintenance requirements, and relapse rates after discontinuation are unknown (7).
6. Population heterogeneity: Studies often mix clinically distinct populations (e.g., high-altitude insomnia vs. chronic insomnia disorder, PTSD-related vs. primary insomnia), limiting generalizability.
7. Publication bias: Negative trials are likely underrepresented in the current literature.

### 4.4 Knowledge gaps and research priorities

Multiple critical questions require investigation before tVNS can be considered an evidence-based sleep medicine intervention. Priority research needs include: (i) standardized, dose-finding RCTs to optimize stimulation parameters per disorder; (ii) head-to-head trials vs. established first-line treatments; (iii) multi-center RCTs with adequate power (*n* > 200 for insomnia, *n* > 50 for RLS); (iv) sham-controlled RCTs for pharmacoresistant RLS; (v) long-term follow-up studies (≥12 months); (vi) identification of predictive biomarkers (baseline HRV, inflammatory markers, polysomnographic phenotyping) (25); (vii) mechanistic substudies validating proposed pathways; and (viii) cost-effectiveness and real-world implementation studies (26).

### 4.5 Clinical implications and recommendations

Based on current evidence, and acknowledging the limitations outlined above, the following tentative recommendations can be proposed. For chronic insomnia disorder, tVNS represents a potentially useful complementary non-pharmacological option for patients who decline or are intolerant to CBT-I, pending larger multi-center replication — it should not replace CBT-I as first-line therapy (7–9). For severe pharmacoresistant RLS, cymba concha taVNS (2 Hz) may be considered as an experimental option in specialized centers when dopaminergic therapy fails or produces intolerable side effects, provided patients are informed of the preliminary level of evidence (14, 15). For OSA, tVNS is not recommended as a treatment for sleep-disordered breathing; clinicians must clearly distinguish auricular tVNS from invasive cervical VNS, which unequivocally worsens OSA (12, 13, 17). For PTSD-related sleep disturbance and all other sleep disorders, available data are insufficient to support any clinical use outside of research settings (27).

## 5 Conclusion

Transcutaneous auricular vagus nerve stimulation demonstrates emerging, but preliminary, promise as a non-invasive neuromodulatory approach for selected sleep disorders. The strongest available evidence supports potential applications in chronic insomnia disorder and pharmacoresistant restless legs syndrome, based respectively on three sham-controlled RCTs and two prospective open-label studies. For OSA, the available evidence relates exclusively to invasive cervical VNS, which causes harm, and no human tVNS data support clinical application. For PTSD and other sleep disorders, evidence remains insufficient.

The field is limited by small sample sizes, marked protocol heterogeneity, short follow-up, and the absence of active comparators and sham-controlled RCTs for RLS. Well-powered, multi-center randomized trials with active comparators, extended follow-up, mechanistic substudies, and standardized stimulation protocols are essential. Identification of treatment responders through baseline biomarkers and investigation of tVNS as a complement to established therapies represent key priorities. With appropriate evidence development, tVNS may eventually emerge as a valuable addition to the sleep medicine armamentarium.
